# Comparative Study of the Pharmacological Properties of Luteolin and Its 7,3′-Disulfate

**DOI:** 10.3390/md20070426

**Published:** 2022-06-28

**Authors:** Emma P. Kozlovskaya, Aleksandr M. Popov, Olga N. Styshova, Aleksey I. Vakhrushev, Tatyana A. Rutckova, Anna B. Podvolotskaya, Ludmila A. Tekutyeva

**Affiliations:** 1 G.B. Elyakov Pacific Institute of Bioorganic Chemistry, Far Eastern Branch of the Russian Academy of Science, 159 Prospect 100-Letiya Vladivostoka, Vladivostok 690022, Russia; popovam@piboc.dvo.ru (A.M.P.); krivoshapkoon@mail.ru (O.N.S.); va78834@gmail.com (A.I.V.); tanya1119@yandex.ru (T.A.R.); 2 Department of Bioeconomy and Food Security, School of Economics and Management, Far Eastern Federal University, Vladivostok 690922, Russia; apodvolot7777@mail.ru; 3 ARNIKA, Territory of PDA Nadezhdinskaya, Volno-Nadezhdinskoye 692481, Russia

**Keywords:** luteolin (LT), luteolin 7,3′-disulfate (DSL), flavonoids, sulfated flavonoids, pharmacological activity

## Abstract

The global spread of the metabolic syndrome, oncological and viral diseases forces researchers to pay increased attention to the secondary metabolites of marine hydrobionts, which often have a high therapeutic potential in the treatment of these pathologies and are effective components of functional food. The flavone luteolin (LT), as one of the most widely distributed and studied plant metabolites, is distinguished by a diverse spectrum of biological activity and a pleiotropic nature of the mechanism of action at the molecular, cellular and organismal levels. However, there is still practically no information on the spectrum of biological activity of its sulfated derivatives, which are widely represented in seagrasses of the genus Zostera. In the present work, a comparative study of the pharmacological properties of LT and its 7,3′-disulfate was carried out with a brief analysis of the special role of sulfation in the pharmacological activity of flavonoids.

## 1. Introduction

Flavonoids are low-toxic, natural compounds that have a variety of biological activities [1,2,3,4,5]. They regulate various signaling pathways in cells, playing an important role in the prevention and treatment of pathologies associated with inflammation (atherosclerosis, metabolic syndrome, diabetes mellitus, immunological, oncological and neurodegenerative diseases) [1,2,3,4,5,6,7,8,9,10,11]. Flavonoids have an antispasmodic effect, including on the vessels of the heart and brain, have a positive effect on metabolic processes in the myocardium, have an antiarrhythmic effect, inhibit platelet aggregation and their adhesion to the epithelium of the vascular wall, and normalize blood rheology and carbohydrate metabolism, providing, first of all, anti-inflammatory and immunomodulatory action [12,13].

**Figure 1:**
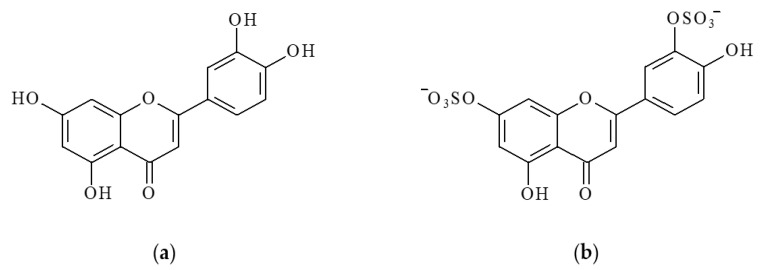
Structural formulas of luteolin (LT) (**a**) and luteolin 7,3′-disulfate (DSL) (**b**).

Luteolin (LT), a water-insoluble 3′,4′,5,7-tetrahydroxyflavone, is one of the best-studied bioflavonoids (Figure 1a). Studies of the biological effects of LT using various animal models and in humans have revealed a variety of beneficial properties of this bioflavonoid. LT, changing the activity of various enzymes involved in metabolism, target receptors and signal transduction pathways, has a wide range of therapeutic effects, including cancer, atherosclerosis and diabetes mellitus [6,7,8,9,10,11,12,13]. It should be noted that no serious side effects have been registered with its use so far [14].

LT transforms into water-soluble metabolites (glucuronides and glycosides) by liver and intestinal epithelial cells in the human body [7]. It is known that natural analogs of glycosylated and glucuronide derivatives of the LT are characterized by high bioavailability for intestinal epithelial cells [15] and also have antioxidant, antibiotic, antiviral, antitumor, cardiovascular, antidiabetic, antiallergic, anti-inflammatory and immunomodulatory effects [16,17,18].

Sulfation is another important metabolic pathway for LT in both plant and animal tissues. Luteolin sulfates are widely distributed in higher land plants [19,20,21,22]. In marine plants, they are found in seagrasses of the family Zosteraceae (*Zostera marina* and *Z. asiatica*) [23,24,25,26,27] and Hydrocharitaceae (*Thalassia testudinum*) [11]. One such sulfated derivative of LT is luteolin 7,3′-disulfate (DSL) (Figure 1b).

In the form of sulfated derivatives, LT circulates in the blood plasma and penetrates various cells of the tissues of the human body. At the same time, sulfated LT is more efficiently absorbed by the epithelium than LT [20]. Unlike LT, the medical and biological activity of sulfated forms of LT has not been practically studied up to now. Therefore, experimental testing of the pharmacological properties of sulfated derivatives of LT seems natural and justified.

The present study is dedicated to a comparative study of the therapeutic and prophylactic use of DSL, using experimental models of pathological conditions associated with hyperlipidemia, diabetes, tumor growth, toxic hepatitis and tick-borne encephalitis.

## 2. Results

### 2.1. Antioxidant Activity

**Figure 2:**
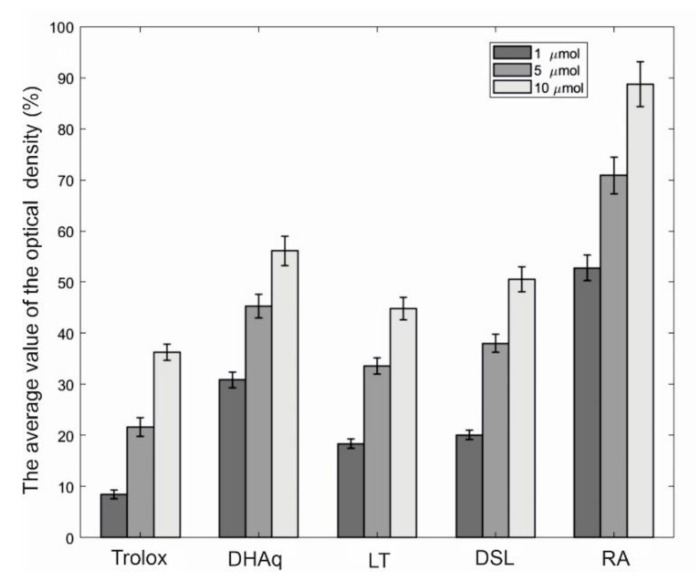
Comparative study of the protective activity of bioflavonoids against linoleic acid peroxidation. The horizontal axis indicates substances and their concentrations (μM), and the vertical axis indicates the inhibition of linoleic acid peroxidation, expressed as % of the control. The average value of the optical density in the control is 0.242 ± 0.016 (100%). The results of three experiments (1–10 µmol) performed in five replicates are presented as m ± SE (Standard Error). Abbreviations: DHAq—dihydroquercetin; LT—luteolin; DSL—luteolin 7,3′-disulfate; RA—rosmarinic acid.

The comparative study of the protective activity of a number of bioflavonoids against linoleic acid peroxidation is shown in Figure 2. It can be seen that rosmarinic acid (RA) in this test system manifests itself as the most effective antioxidant, while LT and DSL are slightly inferior in terms of the effectiveness of the protective action of dihydroquercetin (DHAq). When compared with trolox, the studied substances can be arranged in a descending order according to the effectiveness of preventing the oxidation of linoleic acid: RA > DHAq > DSL > LT > Trolox.

### 2.2. Safety Assessment of Investigational Products

The general toxic properties of the DSL and LT were evaluated during their intragastric administration. DSL, as well as LT, demonstrates acute toxicity at doses higher than 500 mg/kg. The study of subchronic toxicity shows that the intragastric administration of drugs at a dose of 50 mg/kg every day for 10 days did not cause noticeable changes in the biochemical parameters of blood plasma relative to animals that did not receive the drug under study (intact control).

### 2.3. Hepatoprotective Activity

Our results (Table 1) indicate that DSL more effectively protects liver cells from the toxic effect of a strong liver poison (carbon tetrachloride) than LT, which is an effective protective agent in hepatic pathologies of various etiologies [23,24].

### 2.4. Antidiabetic Activity

As the experiment showed, the increase in glucose in the group of animals with induced alloxan diabetes in comparison with the intact group increased by about 2.5 times. DSL and LT at doses of 0.5 to 2.0 mg/kg reduced glucose levels by at least 20% compared with the negative control. The most effective in this experiment was DSL at a dose of 0.5 mg/kg (30% reduction) (Table 2).

The introduction of alloxan reduced the weight of the pancreas by at least 20%. The therapeutic use of DSL protects this organ. At the same time, the protective activity of DSL was most effective at a dose of 0.5 mg/kg. LT in its protective effect on the pancreas (judging by its weight) is not inferior to DSL. The concentration of bilirubin increased by approximately 20% in the group of animals with induced alloxan diabetes (negative control). DSL at a dose of 0.2 mg/kg reduced its content to normal values (group of intact animals). LT in the studied concentration range did not have a normalizing effect on this indicator. In the group of animals with alloxan diabetes (negative control), an increase in the content of thiobarbituric acid-reactive products (TBARs) by approximately 50% was observed. DSL at a dose of 0.1 and 0.2 mg/kg reduced the level of TBARs approximately twice, compared with the group of intact animals. The protective effect of LT on this indicator, which was used in doses 10 times higher (1.0 and 2.0 mg/kg), was similar to the effect of DSL.

Therefore, it can be concluded that DSL has a more pronounced therapeutic effect in experimental diabetes induced by alloxan than LT.

### 2.5. Cardioprotective Activity

The data of a comparative analysis of the effect of various concentrations of LT and DSL in the model of experimental hyperlipidemia are given in Table 3. It can be seen that LT and DSL in the studied concentration range have a modulating effect on the level of triglycerides (TG) and total cholesterol (Ch) in the blood plasma of animals. The nature of the effect of DSL on this indicator largely depends on the dose used.

It should be noted that DSL at a dose of 1.0 mg/kg sharply reduced (by about two times) the concentration of atherogenic VLDL cholesterol in the blood of animals in relation to the negative control. The calculation of the atherogenic index showed that DSL in the studied concentration range was much more effective than LT and thus reduced the risk of developing cardiovascular diseases. Both DSL and LT at a dose of 1.0 mg/kg had little effect on the content of HDL-C.

A comparison of the drug’s effect on the activity of blood transaminases shows that in the studied range they have little effect on this indicator. The calculation of the De Ritis ratio shows that this indicator fluctuates within the normal range. It should also be noted that DSL and LT in the studied concentration range reduced the content of malondialdehyde (MDA) in blood plasma by more than two times, while DSL at a dose of 1.0 mg/kg was more than two times more effective than LT. Therefore, DSL, a water-soluble form of the well-known flavone LT, has a pronounced preventive effect in experimental disorders of lipid and cholesterol metabolism caused by the widely used inducer Tyloxapol. Recently, a possible mechanism for the therapeutic action of DSL has become clear. Using the data of an optical biosensor of surface plasmon resonance, spectral titration, analysis of Cd values and enzymatic activity, it was determined that DSL inhibits the activity of one of the key enzymes in the pathway of cholesterol biosynthesis, lanosterol 14-alpha-demethylase (cytochrome P450 (51), CYP51A1), more selectively in its comparative testing with other structural analogues LT and baicalein. One of the binding sites for DSL to CYP51A1, distant from the heme, was predicted by molecular docking. It has been shown that DSL interacts not with the hydrophobic C-terminal of the F-helix but with the site of binding of lanosterol to the active site of CYP51A1, which ultimately leads to the effective blocking of cholesterol biosynthesis [28]. Therefore, evidence was presented for a possible molecular mechanism of the therapeutic corrective action of DSL in pathological disorders of cholesterol metabolism and positioning it as a new modulator of CYP51A1 activity.

### 2.6. Anti-inflammatory Activity

Carrageenan-induced paw edema is one of the most popular tests used in the screening of biophenols and has long established itself as a valid model for the study of new anti-inflammatory drugs [29].

The data in Table 4 shows that the anti-inflammatory effect of DSL is approximately 1.5 times higher than that of LT. Thus, DSL has a higher anti-inflammatory effect than LT.

It can be seen that the drug “Indomethacin”, taken as a positive control, demonstrates the highest anti-inflammatory activity. The study drug DSL is approximately two times less effective than the drug “Indomethacin”. However, the anti-inflammatory effect of DSL is approximately 1.5 times higher than that of RA and more than 3 times higher than that of LT. DSL appears to be a promising molecule for the development of topical formulations and systemic agents against inflammatory skin diseases.

### 2.7. Comparative Evaluation of the Effect of Ointments on the Course of Experimental Allergic Contact Dermatitis

For the treatment of contact dermatitis induced by 2,4-dinitrofluorobenzene, ointment preparations containing DSL, LT and an antihistamine preparation in the form of Sinaflan ointment were used. An analysis of the course of experimental dermatitis in animals shows that already on the first day after a single skin application of a dinitrofluorobenzene solution, moderate hyperemia appeared in all control animals. After each subsequent application of the allergen, the severity of local changes aggravated, and by the 9th-11th day of the disease, sharp damage to the skin was noted with the formation of a deep hemorrhagic crust. An increase in temperature, the sharp swelling of tissues, lethargy and the inhibition of the motor activity of the animals were also noted.

As can be seen from the data shown in Table 5, the treatment of experimental animals with ointments led to a complete or partial restoration of the initial parameters of the skin by the 13th day of the experiment. In mice, moderate hyperemia and edema were observed, which disappeared at the end of the experiment (day 13), while in the control group of comparison, acute inflammatory phenomena prevailed during this period. Indices of reduction in the severity of skin manifestations ranged from 72.2 to 82.2% (in the case of applying ointments).

It is known that LT can suppress inflammatory mediators (for example, IL-1β, IL-6, IL-8, IL-17, IL-22, TNF-α and COX-2) and regulate various signaling pathways (for example, NF-kB, JAK-STAT, as well as TLR), that is, gently modulate many immunological and inflammatory processes in the skin [30]. Obviously, therefore, the proposed experimental formulations of ointments for the treatment of dermatological disorders containing DSL and LT on a lanolin basis significantly exceed the therapeutic activity of the positive control (the antihistamine drug in the form of Sinaflan ointment). In addition, experimental ointments additionally demonstrate specific anti-allergy activity, which is expressed in a decrease in the severity of contact dermatitis treatment.

### 2.8. Antitumor Activity

Despite the extensive study of LT as a potential antitumor agent, there is no information in the literature about the exact mechanisms by which it inhibits cancer progression. However, there is no doubt that LT prevents tumor development to a large extent due to the inactivation of various signal biochemical transcription pathways necessary for the growth and metastasis of tumor cells [31].

When studying the antitumor activity of DSL, LT and cyclophosphamide (CP) (positive control) using the model of the ascetic variant of Ehrlich’s tumor, it was shown that CP at a dose of 100 mg/kg with a single intraperitoneal injection increased the average life expectancy by 6.2% compared with the control. DSL and LT at oral administration at a dose of 10 mg/kg and five-time intraperitoneal administration of DSL, as well as LT, did not show antitumor activity.

A different picture was observed in the study of antitumor activity with the subcutaneous injection of these drugs on the model of the Ehrlich solid tumor (Table 6). As the experiment showed, LT (66.12%) had the highest activity (ITG, in %), which exceeded the antitumor effect of CP (58.69%) and DSL (52.76%). Combination therapy with CF, both with LT and DSL, did not show a synergistic effect.

Therefore, both LT and DSL have moderate antitumor activity comparable to CP when administered subcutaneously in the Ehrlich solid tumor.

### 2.9. In Vitro Antiviral Activity against Tick-Borne Encephalitis (TBE) Virus

An evaluation of the effect of LT and DSL on the initial stages of virus–cell interaction showed that these drugs protected infected transplantable culture of porcine embryonic kidney cells (PEKC) from the cytopathic effect of the TBE virus to varying degrees. Data on the study of the effect of LT and DSL on the reproduction of the TBE virus, presented in Table 7, indicate that their use leads to a significant decrease of virus replication. It was found that at concentrations from 0.01 to 1 µg/mL, LT and DSL reduced the accumulation of the virus by 3. 0–4.0 lg TCID50/mL (fifty percent tissue culture infective dose) compared with the control. At lower drug concentrations (0.001 and 0.0001 µg/mL), the antiviral activity of DSL was higher than that of LT by 2 lg TCID50/mL (*p* < 0.05).

Previously, LT was shown to effectively inhibit the cytopathic effect of the influenza virus (H3N2) in vitro [3]. It is known that 7-rhamnoside quercetin has a pronounced antiviral activity in vitro against an epidemic virus that causes diarrhea in pigs. In terms of this activity, quercetin 7-rhamnoside exceeded quercetin and LT by more than 10 times. The action of this water-soluble metabolite is not associated with the inhibition of the absorption of the swine diarrhea virus but with the suppression of the early stages of its replication. We found that DSL had been more effective than LT in suppressing the in vitro replication of a highly pathogenic TBE virus strain at the early stages of infection. When evaluating the protective effect of various doses of LT and DSL (from 3.25 mg/kg to 25 mg/kg) and methods of its administration in vivo, it was found (Table 8) that the drug had a dose-dependent protective effect and increased the average lifespan of infected animals. Therefore, in the control group of mice infected with the TBE virus at a dose of 100 LD50, mortality was 100% on the 10th day of observation, and life expectancy was 8.2 ± 0.81 days. At the same time, among animals infected with the same high dose of the virus and treated with the drugs, the highest percentage of survivors was observed in the group of mice with an intraperitoneal injection of 6.25 mg/kg (35.0 ± 5.8%) and 3.25 mg/kg (42.5 ± 9.6) of LT and DSL (respectively).

Among animals treated with DSL, the most survivors (55.0 ± 5.8% and 57.5 ± 5.0%) were observed when the drug was administered orally at doses of 25 and 12.5 mg/kg respectively. By the end of the final observation period (21 days), it was concluded that LT and DSL preparations contributed to an increase in the averagelife span of animals. Therefore, life expectancy in mice treated with LT 12.5 mg/kg orally was 11.4 ± 1.12 days, which increased life expectancy by 39% compared with the control group (8.2 ± 0.81 days). At the same time, the oral administration of 12.5 mg/kg DSL protected 36.7 ± 5.8% of infected animals from death, where life expectancy was 13.1 ± 0.86 days, which increased their average life expectancy by 60% compared with those the same in the control group. We found that the oral administration of DSL at a dose of 12.5 mg/kg effectively protected mice infected with a highly pathogenic TBE virus strain from death.

It has been shown that DSL is superior to the reference drug LT in terms of protective effect. The data obtained in this work allow us to recommend DSL for further trials, especially in combination therapy.

## 3. Discussion

The comparative study analysis of LT and DSL pharmacological properties shows that DSL is often more effective than LT under in vivo conditions. Its solubility in biological fluids plays an important positive role in the pharmacological activity of DSL. Over the past ten years, much attention has been paid to the bioavailability of polyphenolic compounds. The measurement of the levels of conjugated and unconjugated polyphenols in plasma, urine and tissue samples provides important information not only on the distribution of absorbed substances but also on their metabolic profiles during absorption and circulation in the blood. In particular, the effective concentrations of flavonoids and their conjugates in blood plasma are more important indicators than the effective concentrations that are recorded in in vitro experiments. Studies have shown that the penetration of polyphenols into blood plasma is extremely low and reaches maximum concentrations of less than a few micromoles, which often contradicts effective doses obtained in experiments in vitro. Most in vitro experiments require much higher concentration of polyphenols (greater than 5–10 µmol) to demonstrate a biological effect. When taken orally, any drug goes a long way: first, it is metabolized by enzymes of both the bacterial microflora and the gastrointestinal tract, penetrates through the mucous membrane, is absorbed by intestinal epithelial cells, enters the blood of the mesenteric system, passes through the liver, and then reaches the systemic circulation and, finally, interacts with tissue or cellular target receptors and has a pharmacological effect. Most studies have found that the vast majority of flavonoids that enter the human body with food are modified by intestinal and liver epithelial cells [32]. For example, resveratrol (RV) and LT, when administered to animals and humans, are registered in blood plasma as glucuronide and sulfated derivatives [33,34,35]. Storniolo and Moreno examined the antioxidant activity and the antiproliferative action of three RV metabolites on human colorectal cancer cell line cultures [33]. They showed, for the first time, that RV metabolites remain active after their biosynthesis, contributing to the health benefits previously attributed to RV alone. This fact casts doubt on the pharmacological significance of the results of the study of LT in vitro and indirectly testifies in favor of the fact that most of the biological effects of LT observed in vivo are characteristic of its metabolites.

The study of the pharmacological properties of flavonoids indicates a low proportion of aglycones in the composition of metabolites circulating in the blood; however, many researchers still use aglycones in in vitro experiments to study the mechanisms, which are often not even found in blood plasma [14,16]. This raises the question of the adequacy of numerous in vitro data on the biological activity of flavonoids.

At the same time, the spectrum of biological activity, both in vivo and in vitro, of glucuronides and flavonoid sulfates, as well as other metabolites that are actually present in the blood, remains poorly studied. Therefore, at present, it is of particular relevance to conduct studies of the pharmacological activity of flavonoid metabolites [36].

It has been established that sulfated LT is more efficiently absorbed by epithelial cells than LT [16]. It cannot be ruled out that sulfated LT conjugates, such as estrone sulfate, serve as an inactive pool of flavonoids and, upon hydrolysis by sulfatases, reach target tissues [37]. The very low bioavailability of pure flavonoids is due to its extremely active metabolism in the intestinal wall and in the liver, i.e., its biotransformation, mainly into glucuronides and LT sulfates. Consequently, the vast majority of LT in these modified forms reaches the receptor and causes a biological (pharmacological) effect [38,39].

It has been shown that organic anions such as sulfated phenol conjugates, in particular sulfated derivatives of LT, with the assistance of organic anion transporters and under the action of a strong proton gradient, have an increased ability to penetrate into the epithelial cells of the intestine and then be quickly transported into the blood plasma [39].

Studies have shown that sulfated derivatives of LT, in particular DSL isolated from the seagrass *Z. marina*, are significantly more effective than LT and may be worthy candidates for clinical trials.

## 4. Materials and Methods

### 4.1. Preparations

DSL was isolated from the seagrass *Z. marina* according to original technology [27]. The DSL structure was confirmed by elemental and NMR analysis (^13^C and ^1^H), mass spectrometry and UV and IR spectroscopy. LT from Sigma (Sigma-Aldrich, St. Louis, MO, USA) was used as a reference drug.

### 4.2. Antioxidant Activity in Linoleic Acid Peroxidation

Linoleic acid peroxidation was assessed by the ferrothiocyanate method according to [40]. A solution containing 1 mL of each sample, 2 mL of 2.5% linoleic acid in ethanol, 4 mL of 0.02 M phosphate buffer (pH 7.0), 0.4 mL of 100 mM 2,2′-azobis (2-methylpropionamidine) dihydrochloride and luminol and 2 mL of distilled water was mixed and placed in a thermostat at 37 °C for 3 h. Aliquots of 0.1 mL were taken from the reaction mixture after 24 h and diluted with 9.7 mL of 75% ethanol, then with 0.1 mL of 30% aluminum thiocyanate solution and, finally, 0.1 mL of 0.02 M FeCl2 in 3.5% hydrochloric acid. The red absorbance was measured at 500 nm after 24 h, when it reached the maximum value in the control. The results of three experiments (1–10 µmol) performed in five replicates are presented as m ± SE (Standard Error).

### 4.3. Experimental Animals

CBA, CD-1 and BALB/c mice (20 ± 2 g) were used as a test system for studying the activity of natural substances. The experiments were performed on animals purchased from the laboratory animal nursery “Pushchino” and bred in the vivarium of the Pacific Institute of Bioorganic Chemistry of the Far Eastern Branch of the Russian Academy of Sciences (PIBOC FEB RAS) (certificate available).

The animals were kept in accordance with the GOST 33216-2014 “Guidelines for accommodation and care of animals. Species-specific provisions for laboratory rodents and rabbits”. After the experiment completion, they were subjected to euthanasia. Studies using experimental animals were carried out in accordance with the GOST 33044-2014 “Principles of good laboratory practice” and “Guidelines for conducting preclinical studies of drugs”, edited by Mironov et al. [41]. All experiments were approved by the Ethical Committee for Animal Research of the PIBOC FEB RAS, protocol code 08/19, date of approval 27.03.2019.

### 4.4. Determination of Biochemical Parameters of Blood

The sampling of biological material from animals was carried out over 14 h of fasting. The determination of biochemical parameters in blood plasma was made using standard diagnostic kits (Olvex-Diagnostikum, St. Petersburg, Russia), and the optical density of the solutions was determined on a 1000 series CE 1021 spectrophotometer (Cecil Instrumentation Services Ltd., Cambridge, UK).

### 4.5. Determination of TBARs

The method is based on the formation of a colored complex in the interaction of MDA with thiobarbituric acid (TBA). First, 50 µL of blood plasma was diluted in 575 µL of water, and 250 µL of 17% trichloroacetic acid were added. The formed precipitate was separated by centrifugation for 10 min. at 3000 rpm. To 1 mL of the supernatant, 350 µL of saturated, filtered TBA was added, and the samples were placed for 10 min. in a boiling bath. Samples containing distilled water instead of the supernatant were used as controls. After the development of a pink color, the samples were cooled to room temperature. The optical density was measured at 520 nm [42]. The calculation of the TBARs level was carried out according to the formula:

(1) $$C\;(\mathrm{mmol}/L)=“\Delta E”/(1.56\times 10^{5}),$$

where C is the amount of TBARs in blood serum; ΔE is the difference between the extinctions of the experimental and blank samples; and 1.56 × 10^5^ is the molar extinction coefficient.

### 4.6. Definitions of Acute and Subchronic Toxicity

The study of the acute toxicity of the studied substances was carried out on CBA mice weighing 20–22 g prior to the start of the study; the external condition of the animals was examined. Animals with abnormalities detected during the examination were not included in the experiment. Furthermore, all animals were randomly divided into control and experimental groups of 8 animals each and kept for 7 days in their cages to acclimatize to laboratory conditions. The general toxic properties of the studied substances were evaluated during their intragastric administration for 14 days after a single injection. The assessment of subchronic toxicity was carried out with the intragastric administration of the test substances for 10 days, according to Mironov et al. [41]. The study was carried out on mice of the CBA line weighing 20–22 g. The number of animals in each group was at least 8. The analysis of the effect of the drug was evaluated within 14 days (observation of the animals should be at least 14 days after a single injection of the test substance).

### 4.7. Toxic Hepatitis Model

The determination of the hepatoprotective properties of DSL and LT was carried out on a model of toxic hepatitis induced by carbon tetrachloride (CCl_4_) in experimental animals (pathogen-free CD-1 mice weighing 20 ± 2 g) [43]. The preventive effect of drugs was studied in 4 groups of mice (8 individuals in each). Two experimental groups of mice were administered drugs at a dose of 2 mg/kg for 4 days. Two control groups of animals, intact animals and a negative control, received distilled water. Then, 16 h after the last drug intake, one control group of animals (negative control) and two experimental groups of animals were intraperitoneally injected with a mixture of CCl4-vegetable oil (1:1, by volume), 300 μL per 100 g of animal body weight. Two hours after CCI_4_ intoxication, the animals were euthanized. Heparinized blood was centrifuged at 4000 rpm for 15–20 min. The resulting blood plasma was used for biochemical studies.

### 4.8. Experimental Models of Alloxan Diabetes

The studies were carried out on male mice of the CBA line weighing 20–22 g. Alloxan dissolved in a sterile saline solution was administered to mice after a 16-h fast at a dose of 200 mg/kg. Seventy-two hours after an overnight fast, blood glucose levels in the mice were determined using a Satellite glucometer (Elta, Moscow, Russia). Mice with blood glucose levels higher than 11.1 mmol/L were classified as diabetic mice and taken for further research. Experimental animals were divided into the following groups of 8 individuals in each: (1) intact, (2) diabetic (negative control) and (3) diabetic, which were administered the test substances for 15 days after the induction of alloxan diabetes.

LT and DSL were administered orally at doses of 1 and 2 mg/kg and 0.1, 0.2 and 0.5 mg/kg, respectively. After the end of the experiment, blood samples were taken, and the main indicators of changes in carbohydrate metabolism were determined in the glucose tolerance test using a Satellite glucometer (Elta, Moscow, Russia) 60 min after the oral administration of glucose at a dose of 4 g/kg of body weight.

### 4.9. Experimental Models of Hyperlipidemia

The modeling of hyperlipidemia was carried out on male CBA mice weighing 18–20 g and kept under normal vivarium conditions. Animals were subdivided into the following groups of 8 individuals in each: (1) intact, (2) hyperlipidemia and (3) hyperlipidemia with 3-fold administration of the studied preparations every day (prophylactic effect of substances). The development of a picture of pronounced hyperlipidemia was ascertained 24 h after a single injection of the drug “Tyloxapol” at a dose of 200 mg/kg of body weight. After the end of the experiment, blood samples were taken, and the main indicators of changes in lipid metabolism (triglycerides (TG), total cholesterol (Ch) and cholesterol of high-density lipoproteins (HDL)) were determined. The level of cholesterol of low-density lipoproteins (LDL) was calculated using the well-known Friedwald formula:

(2) LDL=Ch–HDL–TG/2.2,

where LDL—cholesterol of very-low-density lipoproteins; Ch—cholesterol; HDL—cholesterol of high-density lipoproteins and TG—triglycerides.

The atherogenic index (AI), which serves as an assessment of the risk of developing atherosclerosis, is calculated using the formula:

(3) AI=(Ch–HDL)/HDL,

where AI is the atherogenic index; Ch is the cholesterol and HDL is the cholesterol of high-density lipoproteins, where the norm is AI ≤ 3 conventional units. There is a threat of atherosclerosis if AI is more than 3.

### 4.10. Carrageenan Model of Inflammation

Nonspecific local inflammation was induced by administering 1 mg of delta-carrageenan type IV (Sigma-Aldrich, St. Louis, MO, USA) in saline (100 µL) to the pad of the hind paw of pathogen-free 8 CBA mice weighing 19–22 g. The anti-inflammatory effect of the studied drugs was evaluated at a dose of 2 mg/kg of body weight. The drug was administered intraperitoneally 1 h before the induction of inflammation by carrageenan. The control group of animals was injected with saline. After 5 h, the mice were euthanized; the paws were cut off along the protrusion of the bone below the articulation of the small and tibia bones and weighed on an analytical balance with an accuracy of 4 decimal places. The level of retardation of granuloma tissue growth was calculated by the equation:

(4) Inflammation slowdown (%)=(Tc − Tm/Tc) × 100%,

where Tc is the weight of granuloma tissue in the control group; Tm is the weight of granuloma tissue in the group that received anti-inflammatory drugs.

### 4.11. Experimental Models of Antitumor Activity

When testing the antitumor activity of the studied substances, Ehrlich ascites and solid tumor were used, transplanted into a pathogen-free line of CD-1 mice weighing 21 ± 2 g (8 individuals in each group). Transplantation, the maintenance of strains and evaluation of the results obtained were carried out as described previously [44,45]. The treatment of animals with tumors was started one day after the transplantation of 3 × 10^6^ ascites or 5 × 10^6^ tumor cells, respectively. The drug was administered orally for 5 days at a calculated dose of 10 mg/kg.

The effect of treatment of animals with Ehrlich’s ascitic tumor was evaluated by the average life expectancy (ALE, %) compared with the control using the formula:

(5) ALE (%)=(Td/Cd)×100%,

where Td and Cd are the average values (days) of the death of animals in the experimental and control groups, respectively.

The effect of treatment of animals with Ehrlich’s solid tumor was assessed by the inhibition of tumor growth (ITG, %) compared with the control using the formula:

(6) ITG (%)=(1 − w/Cw)×100%,

where Tw and Cw are the average values (mg) of the tumor weight in the experimental and control groups, respectively.

When studying the antitumor activity of DSL, we evaluated its effectiveness both when used alone and in combination with the commercial antitumor agent cyclophosphamide (Kraspharma, Krasnoyarsk, Russia).

The antitumor activity of the DSL was evaluated separately and also in combination with a commercial antitumor agent cyclophosphamide (Kraspharma, Krasnoyarsk, Russia).

### 4.12. Determination of Antiviral Activity

#### 4.12.1. Virus

We used the TBE virus of the Far Eastern subtype, strain P-73, isolated from the brain of a person who died from TBE with a focal form of infection. The infectious titer of this strain on a transplantable culture of porcine embryonic kidney cells (PEKC) was 107 TCID50/0.2 mL in the experimental and control groups, respectively.

#### 4.12.2. Cell Culture

To analyze the antiviral activity of the preparations, line of PEKC (Gamaleya Research Institute of Epidemiology and Microbiology, Moscow, Russia), which is a highly sensitive model of the TBE virus, were used. Cells were cultured in 24-well plastic plates using growth medium 199 and RPMI (in equal proportions) supplemented with 10% fetal bovine serum and 100 U/mL gentamicin. In the experiment, maintenance medium with 1% serum and gentamicin was used.

Evaluation of the Antiviral Effect of Drugs on Cell Culture

A monolayer of PEKC cell culture was infected with 10-fold dilutions of the TBE virus. The test preparations in various concentrations from/up 0.0001 to 1 µg/mL were added 1 h after the cells were infected with the virus. The plates were incubated in a CO2 incubator for 5 days. The antiviral effect of the drugs was calculated from the decrease in the infectious titer of the virus (∆, lg).

#### 4.12.3. Evaluation of the Protective Effect of Drugs in Experimental TBE

Non-inbred white mice (males weighing 12–14 g) were kept under standard vivarium conditions in compliance with the rules and international recommendations of the European Convention for the Protection of Vertebrate Animals.

Mice were infected with the TBE virus (strain P-73) subcutaneously at a dose of 100 LD50 in a volume of 0.2 mL (viral titer during subcutaneous infection was 6.5 lg LD50/mL). DSL and LT at doses of 3.25, 6.25, 12.5 and 25 mg/kg were administered to animals intraperitoneally (*ip*) and/or orally (*per os*) 1 h after infection with the virus and then for 5 days, once a day. The control groups were uninfected mice that received the test drugs as well as TBE virus-infected mice that did not receive the drugs. There were 10 animals in each group. The animals were observed for 21 days. The antiviral efficacy of the drugs used was evaluated according to the requirements set forth in the manual [28]. All experiments were repeated three times.

### 4.13. Statistical and Graphic Processing of Experimental Data

Statistical data processing was carried out using the Biostatistics software package (Version 4.03) (McGraw-Hill Medical Publishing, Berlin, Germany). Student’s *t*-test was used as a parametric test. The difference between the two compared values was considered significant if the probability of their identity was less than 5% (*p* < 0.05).

## 5. Conclusions

Bioflavonoids act as signal regulators of various biochemical pathways in the cells and tissues of the body [4,7,8,9,46]. Flavonoids and their conjugates have unique mechanisms of protective action. Their long-term use may have a beneficial effect in the treatment of inflammatory diseases. However, the search for bioflavonoids whose effectiveness is comparable to that of non-steroidal and steroidal anti-inflammatory drugs has not yet been successful. Experiments have shown that the protective biological activity of DSL is, in many cases, much higher than that of LT. DSL is rapidly transported into the blood plasma of animals and humans through the intestines, bypassing the stages of modification by intestinal and liver cells, which increases its bioavailability and, consequently, the effectiveness of the pharmacological action. We assume that DSL and LT interact with the same target receptors and cause similar pharmacological effects. In the light of our pharmacological data, it can be assumed that the therapeutic and prophylactic use of DSL and other conjugated forms may be more effective than native flavonoids. Thus, the study of molecular mechanisms of action and the development of therapeutic and prophylactic agents based on conjugated forms of PL seem to be quite modern and promising areas of scientific research.

## Figures and Tables

**Table 1:** Biochemical indicators of blood plasma of experimental animals with hepatitis.

Substance/Animal Group (8 Mice in Each)	Intoxication CCl4	TBARs (µmol/L)	Bilirubin (µmol/L)	ALT (mol/L × h)	AST (mol/L × h)
Intact	−	0.19 ± 0.02	32.23 ± 5.25	0.90 ± 0.02	0.17 ± 0.01
CCl_4_ (−) control	+	2.00 ± 0.19	170.09 ± 14.87	3.37 ± 0.19	4.84 ± 0.80
DSL	+	0.64 ± 0.01	70.18 ± 2.21	2.82 ± 0.10	3.77 ± 1.10
LT	+	1.20 ± 0.18	112.93 ± 18.04	3.58 ± 1.06	2.58 ± 1.20

Results are presented as m ± SD (Standard Deviation). Abbreviations: CCI_4_—carbon tetrachloride; TBARs—thiobarbituric acid-reactive products; ALT—alanine aminotransferase; AST—aspartate aminotransferase; LT—luteolin; DSL—luteolin 7,3′-disulfate.

**Table 2:** Biochemical parameters of blood plasma of experimental animals with alloxan diabetes.

Substance/Animal Group (8 Mice in Each)	Relative Weight of the Pancreas (%)	GTT (mol/L)	TBARs (µmol/L)	ALT (µmol/L)	AST (µmol/L)	Bilirubin (µmol/L)
Intact	8.3 ± 0.12 *	6.93 ± 0.73	1.4 ± 0.03	62.4 ± 2.6 *	145.0 ± 5.9 *	44.5 ± 2.6 *
(−) control	7.2 ± 0.15	17.5 ± 1.25	1.6 ± 0.18	67.6 ± 1.5	164.6 ± 13.7	56.6 ± 1.8
DSL (0.1 mg/kg)	7.4 ± 0.18 *	15.7 ± 4.3	0.7 ± 0.05	174.7 ± 59.8 *	137.0 ± 11.8 *	34.5 ± 1.3 *
DSL (0.2 mg/kg)	7.6 ± 0.17 *	14.9 ± 1.85 *	0.5 ± 0.04	83.2 ± 2.6 *	150.9 ± 7.8 *	45.2 ± 1.9 *
DSL (0.5 mg/kg)	8.7 ± 0.04 *	13.8 ± 3.6 *	0.3 ± 0.05	78.0 ± 1.3 *	145.0 ± 7.1 *	61.5 ± 5.6
LT (0.5 mg/kg)	8.7 ± 0.10 *	15.0 ± 5.6	1.4 ± 0.21	119.6 ± 7.3 *	152.9 ± 15.7	60.0 ± 3.2 *
LT (1.0 mg/kg)	8.2 ± 0.09 *	13.5 ± 1.9 *	1.2 ± 0.02	83.2 ± 15.6 *	152.8 ± 2.1 *	55.8 ± 2.2
LT (2.0 mg/kg)	7.7 ± 0.15 *	12.9 ± 5.2 *	0.6 ± 0.03	161.2 ± 28.6 *	147.5 ± 0.9 *	53.2 ± 1.0 *
Glibenclamide (5 mg/kg)	1.0 ± 0.13 *	14.6 ± 0.57 *	0.5 ± 0.01	85.8 ± 5.2 *	135.2 ± 31.4 *	47.9 ± 2.1 *

* significant difference from the (−) control group at *p* < 0.05. Abbreviations: GTT—glucose tolerance test; TBARs—thiobarbituric acid-reactive products; ALT—alanine aminotransferase; AST—aspartate aminotransferase; LT—luteolin; DSL—luteolin 7,3′-disulfate.

**Table 3:** Biochemical parameters of blood plasma of experimental animals in the treatment of hyperlipidemia.

Substance/Animal Group (8 Mice in Each)	TG (mol/L)	Ch (mol/L)	HDL (mol/L)	VLDL (mol/L)	MDA (µmol/L)	ALT (µmol/L)	AST (µmol/L)	Bilirubin (µmol/L)
(−) control	4.9 ± 0.20	4.1 ± 0.96	2.6 ± 0.32 *	4.5 ± 0.11	2.8 ± 0.10	52.0 ± 7.8	162.3 ± 14.8	25.1 ± 5.8
DSL (0.1 mg/kg)	4.1 ± 0.11	3.9 ± 0.05	2.5 ± 0.97	4.0 ± 0.24	1.7 ± 0.98 *	44.2 ± 7.8 *	159.3 ± 11.8	27.9 ± 2.3
DSL (1 mg/kg)	4.1 ± 0.09 *	3.9 ± 0.12 *	2.6 ± 0.05	3.0 ± 1.20	0.9 ± 0.01 *	44.2 ± 2.6 *	179.9 ± 11.8 *	25.4 ± 1.4
LT (0.1 mg/kg)	3.3 ± 0.21 *	2.9 ± 0.08	2.2 ± 0.0 *	3.7 ± 0.02 *	1.1 ± 0.97 *	44.2 ± 7.8	159.3 ± 5.9	30.7 ± 2.2 *
LT (1 mg/kg)	3.8 ± 0.12 *	3.2 ± 0.15	1.9 ± 0.09	3.5 ± 0.55 *	1.8 ± 0.12 *	44.2 ± 15.6	188.9 ± 2.9 *	23.0 ± 2.8
LT (10 mg/kg)	4.3 ± 1.03	3.7 ± 0.77	2.09 ± 0.01	4.0 ± 0.98 *	1.3 ± 0.03 *	41.6 ± 2.6 *	162.5 ± 10.8	19.9 ± 1.1 *

* significant difference from the (−) control group at *p* < 0.05. Abbreviations: TG—triglycerides; Ch—cholesterol; HDL—cholesterol of high-density lipoproteins; VLDL—cholesterol of very-low-density lipoproteins; MDA—malondialdehyde; ALT—alanine aminotransferase; AST—aspartate aminotransferase; LT—luteolin; DSL—luteolin 7,3′-disulfate.

**Table 4:** Anti-inflammatory activity of the studied substances in the mouse carrageenan model.

Substance/Animal Group (8 Mice in Each)	Inhibition of Inflammation (%)
Indomethacin	21.06 ± 3.8
RA	8.97 ± 0.01 *
LT	3.68 ± 0.2 *
DSL	12.75 ± 0.01 *

* significant difference from the animals treated with Indomethacin group at *p* < 0.01. Abbreviations: RA—rosmarinic acid; LT—luteolin; DSL—luteolin 7,3′-disulfate.

**Table 5:** Therapeutic effect of the studied ointments on the process of wound healing.

Substance/Animal Group (8 Mice in Each)	Treatment Time (day)	7th Day	9th Day	14th Day
		Wound Healing (% of Control)	Wound Healing (% of Control)	Wound Healing (% of Control)
Sinaflan (control)	5	11.2 ± 4.5	64.17 ± 2.3	100 ± 1.4 *
Ointment DSL 1%, lanolin	5	70.7 ± 3.6	70.31 ± 2.3	99.43 ± 1.1 *
Ointment LT 1%, lanolin	5	70.5 ± 5.2	65.8 ± 4.3	100 ± 0.98 *

Results are presented as m ± SD (Standard Deviation). * significant difference from the control group at *p* < 0.05. Abbreviations: LT—luteolin; DSL—luteolin 7,3′-disulfate.

**Table 6:** Comparative study of the antitumor activity of LT and DSL on the Ehrlich solid tumor.

Substance/Animal Group (10 Mice in Each)	Dose (mg/kg)/Interval (h) × Number Injection	ITG (% of Control)
CP	20/24 × 5	58.69 ± 5.9
LT	1/24 × 10	66.13 ± 5.8 *
DSL	1/24 × 10	52.77 ± 4.1 *
LT + CF	1/24 × 10; 20/24 × 5	46.65 ± 10.6 *
DSL + CF	1/24 × 10; 20/24 × 5	56.96 ± 9.7

* significant difference from the group of animals treated with CP at *p* < 0.05. Abbreviations: CP—cyclophosphamide, ITG—inhibition of tumor growth; LT—luteolin; DSL—luteolin 7,3′-disulfate.

**Table 7:** Virus-inhibiting effects of LT and DSL in PEKC culture.

Substance	Concentration (µg/mL)	lg TCID50/mL
LT *	1.0	4.0 ± 0.4
	0.1	3.5 ± 0.5
	0.01	3.0 ± 0.4
	0.001	1.0 ± 0.3
	0.0001	0
DSL *	1.0	3.5 ± 0.4
	0.1	3.5 ± 0.4
	0.01	4.0 ± 0.4
	0.001	3.0 ± 0.3 **
	0.0001	2.0 ± 0.3 **
control *	-	7.5 ± 0.5

* all experiments performed in five replicates, ** significant difference from the control group at *p* < 0.05. Abbreviations: TCID50—fifty-percent tissue culture infective dose; LT—luteolin; DSL—luteolin 7,3′-disulfate.

**Table 8:** Virus-inhibiting effect of various concentrations of luteolin and luteolin 7,3′-disulfate in experimental tick-borne encephalitis (TBE).

Substance /Animal Group (10 Mice in Each)	Dose of the Drug (mg/kg)	Method of Drug Administration	10th Day of Experience		21st Day of Experience	
			Protection against Death (%)	Average Life Expectancy	Protection against Death (%)	Average Life Expectancy
Control	-	-	0	8.2 ± 0.81	0	8.2 ± 0.81
LT	12.50	intraperitoneally	32.5 ± 9.6	8.8 ± 1.11	0	
	6.25	intraperitoneally	35.0 ± 5.8	9.0 ± 1.03	0	
	3.25	intraperitoneally	20.0 ± 10.0	8.5 ± 0.56	0	
	25.00	orally	32.5 ± 5.0	9.0 ± 0.89	13.3 ± 5.8	10.5 ± 1.27
	12.50	orally	27.5 ± 5.0	9.0 ± 1.01	16.7 ± 5.8	11.4 ± 1.20
DSL	12.50	intraperitoneally	17.5 ± 5.0	8.3 ± 1.15	0	
	6.25	intraperitoneally	25.0 ± 5.8	9.0 ± 0.98	0	
	3.25	intraperitoneally	42.5 ± 9.6 *	9.0 ± 1.07	0	
	25.00	orally	55.0 ± 5.8 *	9.4 ± 0.87	21.7 ± 6.3 *	12.6 ± 0.95 *
	12.50	orally	57.5 ± 5.0 *	9.4 ± 0.76	36.7 ± 5.8 *	13.1 ± 0.86 *

* significant difference from the control group at *p* < 0.05. Abbreviations: LT—luteolin; DSL—luteolin 7,3′-disulfate.

## Data Availability

Not applicable.
